# Plant tissue culture challenges in Ethiopia and alternative options for low-cost media

**DOI:** 10.12688/f1000research.122627.1

**Published:** 2022-07-26

**Authors:** Kasahun Amare, Geleta Dugassa

**Affiliations:** 1 Applied Biology, Adama Science and Technology University, Adama, Oromia, 1888, Ethiopia

**Keywords:** Alternatives, Biotechnology, Ethiopia, Low-cost

## Abstract

Plant tissue culture (PTC) is the cultivation of any part of a plant in nutritionally defined media under an aseptic and controlled environment, regardless of season and weather. The application of PTC leads to the mass propagation of varietal, high-quality seedlings of ornamental plants, medicinal plants, plantation crops, fruit trees, and forest trees. PTC technology, on the other hand, is more expensive in developing nations, such as Ethiopia, than traditional propagation methods such as seeds, cuttings, grafting, and so on. As a result, it is critical to take steps to cut production costs and explore alternate choices for present PTC obstacles (budget restrictions, procedural and operational matters, and unfortunate interactions and partnerships). In order to lower the unit cost of crop production, cost-effective procedures and the optimal utilization of equipment are required. This can be accomplished by increasing the efficiency of processes and optimizing resource allocation. Gelling agents, macro and micronutrients, equipment, carbon sources, and the utilization of bioreactors, which can minimize space, energy, and labor needs, can all be replaced to lower production costs. Therefore, these alternative options are recommended as a workaround to the problems and are briefly described in this document.

## 1. Introduction

The growth of plant organs, tissues, cells, or protoplasts in specially formulated nutritional media under sterile and well-controlled light, temperature, and humidity conditions is known as plant tissue culture (PTC) ( George *et al*., 2008; Twaij *et al*., 2020). PTC is a remarkable biotechnological tool that has applications in vegetable propagation and improvement, disease elimination, herbicide resistance, salinity tolerance, incorporation of high nutrient content, genetically improved plants, and conservation of endangered plant species, and soon usage of this technology will increase further ( Naik *et al*., 2020; Twaij *et al*., 2020).

With the increasing demand for agricultural, forestry, and horticultural products, the demand for hybrid crops and high-quality, high-yielding, disease-free planting material has increased dramatically in recent decades ( Kadam *et al*., 2018). Due to these factors, the PTC market was valued at USD382.305 million in 2020 and is projected to grow to USD895.006 million by 2030, with a compound annual growth rate of 8.5 percent from 2021 to 2030 ( Srivas & Sumant, 2021).

To meet market demand and provide farmers with planting material, production must be plentiful. However, one of the major disadvantages of PTC is the high cost of culture media (carbon source, gelling agents, and growth regulators), facilities, and high relative humidity inside the culture vessel causes hyperhydricity, somaclonal variation, energy, and labor, which limit the expansion of the techniques and hamper their implementation ( George *et al*., 2008; Chen, 2016; Espinosa-Leal *et al*., 2018).

Due to high manufacturing costs, even small and medium-sized laboratories with limited resources cannot take advantage of the potential benefits of PTC technology ( Naik *et al*., 2020). The poorest countries, including Ethiopia, have missed these opportunities due to a lack of understanding, economic reputation, and legal constraints related to spending ( Getnet *et al*., 2016; Abrar *et al*., 2019). The cost of producing the medium can be as much as 30-35% of the cost of producing micropropagated plants ( Gitonga *et al*., 2010). Electricity costs can account for up to 60% of tissue culture production costs, and labor costs can account for up to 70% ( Espinosa-Leal *et al*., 2018). In addition to high media and others such as inadequate funding and poor management practices, lack of qualified staff, inadequate infrastructure, and poor facilities, staff turnover/brain drain are serious bottlenecks for PTC ( George & Manuel, 2013; Abbebaw *et al*., 2021).

These difficulties have been solved by the development of reliable, inexpensive tissue culture methods that do not compromise plant quality. Chemicals, media, energy, labor, and capital costs are important factors in production. Alternative materials such as gelling agents (isabugol, sago, cassava starch, barley starch, and bulla) can be substituted for agar as a solidifying agent. In addition, carbon sources such as table sugar, potatoes, cane juice, sugar cubes, and brown sugar have already been documented worldwide ( Naik *et al*., 2020). Other possibilities for lowering production cost includes liquid media, reusable glass beads and bioreactors, can be mentioned ( Daud *et al*., 2011; Espinosa-Leal *et al*., 2018). Furthermore, there is a huge demand to develop scale-up systems and automation to reduce the intensive labor need as well as the production cost in PTC ( Aladele *et al*., 2012). In this case, this paper aims to review the current challenges of PTC in the development of various low-cost alternatives in Ethiopia.

## 2. PTCs in Ethiopia and current status

In Ethiopia, biotechnology is covered by seven institutions with a total of 24 branches that deal with various stages of biotechnology research/education and development, including tissue culture, molecular markers, embryo transfer, immunology, vaccines, diagnostic kit development, epidemiology, and genetic diversity. Among those PTC is the most extensively utilized method in agricultural biotechnology ( Abraham, 2009; Kassa, 2011). This valuable training area has been initiated in universities and colleges since the 1980s and 1990s at Addis Ababa University initiated joint research and training programs in biotechnology with Swedish Universities. The programs made it possible to train staff members in agricultural biotechnology and industrial biotechnology and to establish a modest facility ( Wandui *et al*., 2013; Abebaw *et al*., 2021).

Other Ethiopian universities have begun graduate and undergraduate biotechnology training programs, following Addis Ababa University's examples such as Gonder University (undergraduate program); Haramaya University (master’s degree program); Jimma University (master’s degree program), Arba Minch University (master’s degree program), and Bahir Dar University (master’s degree program), Mekelle University and Mekelle Institute of Technology (undergraduate program) and others ( Broek & Rensink, 2021).

However, a broader and more concerted tissue culture research program was instituted by the Ethiopian Institute of Agricultural Research, which in 2000 launched a broader and more coordinated tissue culture research program focusing on micropropagation, optimization of tissue culture protocol, and virus elimination in economically important horticultural crop species, including banana, cardamom, grapevine, citrus, garlic, potato, geranium, enset, coffee, pineapple, black pepper, sweet potato, cassava, Aframomum corrorima and other high-value crops ( Abraham, 2009; Wandui *et al*., 2013; Abebaw *et al*., 2021; Seyoum *et al*., 2021).

Regional agricultural research institutes such as Amhara Regional Agriculture Research Institute (Bahir Dar Tissue Culture Laboratory), Southern Nations, Nationalities and People Region Agricultural Research Institute (Areka Agricultural Research Center), Oromia Regional Agriculture Research Institute (Adami Tulu Agricultural Research Center), and the Tigray Regional Agriculture Research Institute (Mekelle Agricultural Research Center) have made significant advances in tissue culture ( Abebaw *et al*., 2021).

In addition, tissue culture activities are also carried out by private companies; for example, VCI was active until 2017 but was shut down, as was Narus Biotechnology, which appears to have ceased operations. Waginos Biotech is still operational but is more focused on ornamental crops for the local market and small seed potato crops. Tigray Biotechnology Center, Dessie Tissue Culture Center, and Bahir Dar Tissue Culture Enterprise play a role in the commercialization and dissemination of research outputs to stakeholders ( Wandui *et al*., 2013; Abrar *et al*., 2019; Abebaw *et al*., 2021). The most active private plant tissue laboratory is the Bahir Dar PTC, which produces approximately 500,000 plantlets annually ( Broek & Rensink, 2021).

Currently, there is fertile ground in Ethiopia to take advantage of the potential benefits that can come from biotechnology, particularly tissue culture. Some of the micropropagation efforts came to results and commenced to solve the prevailing shortage of planting material ( Seyoum *et al*., 2021). Most production-oriented plant tissue initiatives in the mid-2010s were geared toward expanding large-scale semipublic sugarcane plantations. Most of the seedlings produced in the various laboratories were concentrated in these new plantations; the Tigray Biotechnology Center alone had a production capacity of 40 million, and the Dessie Tissue Culture Center had 20 million plantlets to supply this market ( Abebaw *et al*., 2021; Broek & Rensink, 2021). Between 2009 and 2019, the government had a plan to expand the area covered by state sugar cane cultivation by more than 330,000 hectares and to boost annual sugar production from 2.25 to 4.1 million tons. The program implemented several new sugar plantations, including in the Omo Valley. As a result, Ethiopia increased sugar cane production to 1.5 million tons in 2019. However, limited expansion is currently taking place, putting pressure on PTC labs ( Hamza & Alebjo, 2017; Broek & Rensink, 2021; Seyoum *et al*., 2021).

## 3. The major challenges of PTC

Developing nations, including Ethiopia, are not getting the potential benefits from modern biotechnological tools and products. Even if PTC activities started three decades ago in Ethiopia, particularly in public and private research centers and public universities, they have not reached the commercial level to date ( Getnet *et al*., 2016). Therefore, in the Ethiopian context, this sector is an emerging subject both as an academic subject and as an industrial application with its emerging application prospects. This is evidenced by the introduction of undergraduate courses, funding for new university-level laboratories, and approval for young start-ups in biotechnology sectors ( Hanumanthaiah & Alemu, 2021). A limited investigation and academic institutes in Ethiopia are engaged in basic, advanced and related research areas in biotechnology with limitations in tools, techniques, and management as well as financial constraints ( Abebaw *et al*., 2021; Hanumanthaiah & Alemu, 2021). At the same time, these developments pose challenges for pioneering biotechnological research and development in terms of lack of funding and supplies in government institutions, low management autonomy in maintenance, technical and administrative problems, limited capacities, improper networking and collaborations leading to material and technological results, and financial difficulties ( Hanumanthaiah & Alemu, 2021).

In addition, finding skilled workers for the plant tissue laboratory is the greatest challenge. The work in the labs is tedious and requires dedication. University graduates (Bachelor) do not want to work as normal workers in laboratories. In addition, prices for inputs are increasing; in particular, the chemicals needed to produce the media and growth hormones, equipment cost, and energy sources remain a challenge ( Baidiyavadra *et al*., 2019; Abebaw *et al*., 2021; Broek & Rensink, 2021).

To fully achieve the potential of tissue culture in Ethiopia, answers to these constraints are required. Intervention methods to address these concerns would undoubtedly necessitate a collaborative effort by a number of persons and institutions, which should be backed up by suitable national and regional governments ( Abebaw *et al*., 2021). The Ethiopian government has publicly expressed its interest in the commercialization of agricultural biotechnology as a tool to achieve food security in the country as directed by the prime minister's office to stakeholders ( Seyoum *et al*., 2021). The government recently established a national biotechnology roadmap, which will design and implement policies and strategies for modern biotechnology, prioritizing the agricultural sector. To this end, public universities, research institutes, and some private companies play a promising role in producing a skilled labor force and have started research in biotechnology ( Abrar *et al*., 2019; Abebaw *et al*., 2021). In other cases, alternative options for culture media, such as cane molasses, cane juice, banana extract, and coconut water, are good alternatives to high-quality sucrose to reduce mean costs ( Getnet *et al*., 2016).

### 3.1 Public awareness of PTC

Public awareness is the last step toward the acceptance and application of the technology in practice. Nonetheless, it is believed that the public is generally less supportive of animal biotechnology applications than plant biotechnology applications, possibly reflecting spiritual attitudes and literacy impact (Abbebaw *et al*., 2021). However, the obstacle to plant biotechnology is public acceptance of genetically modified crops, as the technology can be controversial and questionable. Farmers have also expressed their concerns. Farmers are particularly concerned about the requirement for qualified workers to carefully nurture PTC seedlings in nurseries, as well as a variety of other unusual growth procedures that stymie PTC's economic viability ( Bandewar *et al*., 2017).

Therefore, since PTC is a part of plant biotechnology, it is important to be introduced to the young generation to encourage their positive attitudes, which ultimately lead to human capital and public awareness in the field ( Moid *et al*., 2021). In addition, the restriction can be avoided by raising awareness through the organization of training, workshops, national seminars, conferences, demonstrations, and dissemination of the information to the public through television, radio interviews, and other mass media ( Hanumanthaiah & Alemu, 2021).

### 3.2 Inadequacy of funding

High national investments have set the foundation for the development of national and international firms in nations where biotechnology has taken hold. The strong backdrop of national research and development, as well as the introduction of special budgets to stimulate the rise of commercial biotechnology, were two of the key factors for the early birth of biotechnology in industrialized countries. Various governments place a high priority on biotechnology development and provide adequate funding for research and development. For example, Lithuania (29%), Austria (3.3%), Germany (2.8%), and the Russian Federation (1%) spent by the fiscal year of 2020 on the research and development of biotechnology companies. Research and development by biotechnology companies as a proportion of the indicator for the country's research focuses on modern biotechnology ( OECD, 2021).

In FY2019-20, the Ethiopian government funded ETB 0.16 billion for the Ethiopian Institute of Biotechnology, which can be distributed to available departments (Ethiopia’s 2019 Budget document). However, there are public and private biotechnology laboratories in Ethiopia, funded by government and nongovernment sources. Some of the labs are for tissue culture, and others are for molecular biology. Therefore, the fund is not as sufficient to undergo plant biotechnology tasks as needed. Due to fund limitations, research and development carried out by Ethiopia's private sector are limited ( Abebaw *et al*., 2021; Broek & Rensink, 2021). In general, it appears that the level of funding required to get a biotechnology program on track and the level of sustained funding required for subsequent operations are generally underestimated ( Hanumanthaiah & Alemu, 2021).

The available financial resources are still too low to allow countries to engage effectively in cutting-edge activities. While they are coordinated and managed by public research institutions, most laboratories and/or institutions heavily rely on external funding from development partners that may not satisfy our needs. Equally challenging is the low level of private sector participation in biotechnology research and development. In some cases, links with the local private sector are either weak or nonexistent ( Abebaw *et al*., 2021; Broek & Rensink, 2021; Seyoum *et al*., 2021).

Due to the high costs associated with this, long-term planning is required when allocating resources to make optimal use of the infrastructure setup. Medium-term depletion of funds or limited operating budgets was the cause of poor performance, reducing profits to a fraction of what was expected ( Abebaw *et al*., 2021).

Previous studies have proven that there can be no national economic growth without adequate investment in the appropriate technologies. Investment in unsuitable technology is a real solution to major national challenges such as job creation and poverty alleviation. This is obvious in countries that have welcomed and absorbed biotechnology in previous technological revolutions and are now implementing it on a massive scale ( Aladele *et al*., 2012).

Therefore, four separate steps must be recognized in the funding process to enable the full functionality of an established facility. 1) Funding to establish the physical laboratory space large enough to house the extensive instrumentation associated with this equipment-intensive technology. 2) Funding to purchase the critical mass of equipment needed for molecular work. 3) Funding to cover operational costs of expensive reagents, renewable consumables, and technical support. 4) Funding to fund specific research projects for the period necessary to obtain results. With proper financial planning that includes sustainable funding; our labs can become more productive.

### 3.3 Skilled workforce scarcity and knowledge gap

The lack of adequately qualified science, technology, and support personnel severely impedes the application of biotechnology in developing countries, including Ethiopia. Most service personnel do not have the necessary scientific background to use the new technology. Therefore, further training of the staff is urgently required to enable the use of new technologies. The Ethiopian government recently created a national biotechnology roadmap that prioritizes the agricultural sector to design and implement policies and strategies related to advanced biotechnology. To this end, national universities, research institutes, and some private companies play a promising role in developing a skilled workforce ( Abebaw *et al*., 2021; Hanumanthaiah & Alemu, 2021).

PTC is one of the plant biotechnology disciplines that necessitate a time-consuming procedure. 1) medium preparation, dispensing, and discarding; 2) container washing, handling, and transportation; 3) autoclaving; (4) pruning and transplanting of plants and explants; 5) capping and uncapping; 6) removal and disposal of dead and contaminated plants; 7) acclimatization, equipment, and room sterilization; 8) recording of activities and labeling on culture vessels; and 9) surveillance ( Abebaw *et al*., 2021).

In affluent countries, labor is a significant expense at PTC. On the other hand, developing-country labor is often cheaper, which is a significant advantage. Labor accounts for 40% of a tissue culture facility's production costs, 10% for supplies, 20% for overhead, and 30% for sales, general, and administrative operations, according to a typical cost profile ( Datta *et al*., 2017). In the EU, however, labor can account for 60-70% of the cost of in vitro plants ( Savangikar, 2004).

Another bottleneck in PTC is the scarcity of supportive staff for the repair and maintenance of equipment. There is currently a shortage of trained technicians to maintain advanced equipment, so it is necessary to frequently request repairs from foreign manufacturing companies. The challenge after training our workforce is managed to avoid losing our skilled workforce ( Abebaw *et al*., 2021; Hanumanthaiah & Alemu, 2021). This is also the current challenge of PTC laboratories present in Ethiopia. For example, Abebaw *et al*. (2021) reviewed the country's existing laboratories and found a shortage of well-trained and experienced tissue culture personnel and high staff turnover; regrettably, skills for the maintenance and repair of tissue culture equipment are also limited.

Furthermore, many entrepreneurs enter commercial PTCs without sufficient knowledge of tissue culture. To be successful, entrepreneurs must have a basic understanding of the factors that affect plant growth, how plants respond to culture, and how to manipulate plant growth. A solid foundation for plant operations enables entrepreneurs at PTC to change processes to maintain high plant yields. To have such knowledge at PTC, there should be a system for the entrepreneurs to enter a college center. However, in Ethiopia, the number of tissue culture courses offered at public universities is limited ( Seyoum *et al*., 2021).

To overcome the scarcity of manpower, it is advised that there should be a system that encourages students to attend prestigious universities and research centers with scholarships as an important way to gain access to new knowledge. Other forms of collaboration, such as technology transfer and alliances are also needed.

To reduce staff turnover/brain drain, after an efficient recruitment process has been developed to hire the best people, whatever is necessary should be done to retain them. Employees need a living wage to pay for food, shelter, and other basic needs, and this is achieved by providing significant incentives. Attractive incentives can help reduce staff turnover while encouraging employees to be more loyal and productive ( Sawyer, 2019).

### 3.4 Poor facilities

A tissue culture facility can use a variety of low-cost options to simplify operations and cut costs. Equipment and buildings with a preparation room, transfer room, culture or growth room, hardening and weaning area, soil growing area (greenhouses, plastic tunnels), packaging and shipping area, and associated facilities such as an office and chemical storage area, containers, and extras are all physical components of a typical PTC facility. The size of a tissue culture facility's physical components is determined by its functional requirements or production volume ( George & Manuel, 2013).

Ethiopian government-owned PTC laboratories are characterized by adequate infrastructure, and these are a serious bottleneck and were exacerbated by unreliable utility services (electricity and water supply). Furthermore, low efficiency at different steps in the tissue culture process resulted in very low productivity due to the multiplicative effect of the low facility (Abbebaw *et al*., 2021). Therefore, finding a reliable solution to such problems requires adequate funding to provide and maintain adequate facilities to operate. The management of assets and personnel must be aligned to the desired level of production.

### 3.5 Lack of regional technical collaboration linkage

The lack of active biotechnology networks and a regional network may be considered one factor limiting the development of biotechnology in agriculture. To date, research collaborations have usually been established either by individual institutions with specialized laboratories abroad for specific projects or with plant-based networks ( Brook & Mastewal, 2018). Ethiopian entered into a few partnerships with some organizations involved in agricultural biotechnology in a fragmented way; for example, the Open Forum on Agricultural Biotechnology is one of the agricultural research collaborators with Ethiopia and other countries, such as Kenya, Burkina Faso, Ghana, Nigeria, Tanzania and Uganda ( Rock & Schurman, 2020). Another example of partnerships and collaborations in the country is the Addis Ababa University Institute of Biotechnology with the Swedish University of Agricultural Sciences. Networks are built based on common areas of interest; they facilitate the joint development of training programs and research projects, as well as student exchanges and research stays ( Brook & Mastewal, 2018).

There seems to be an urgent need for Ethiopia to join as many large-scale plant-based research networks as possible, particularly those involving other developing countries of comparable economic status and interests in plant research. In addition, the establishment of a regional network is necessary for the sustainable development of biotechnology. Critical to the success of this network would be support for the FAO Global Plant Biotechnology Programme, which aims to strengthen plant biotechnology institutions in developing countries.

Generally, the weak collaborative linkages and/or partnership among the different stakeholders along with the tissue culture development and the delivery pipeline is considered the bottleneck for the advancement of PTC research centers, universities, and private laboratories should create a very good platform and collaboration that enables solving the key challenges ( Seyoum *et al*., 2021).

### 3.6 The challenge of high cost conventional PTC

In high-tech laboratories, PTC has become a standard method of plant propagation. However, as compared to traditional propagation methods such as seeds, cuttings, grafting, and so on, the production costs of conventional tissue culture are quite high ( Suman, 2017). Traditional PTC has been utilized for decades; nevertheless, the expensive cost of tissue culture production is a disadvantage for laboratories with limited resources, especially in developing nations ( George & Manuel, 2013). The high cost of primary production prevents PTC from being adopted on a big scale. As a result, it is critical to take steps to lower production costs. In order to lower the unit cost of crop production, cost-effective techniques and optimal equipment utilization are required. This can be accomplished through increasing the efficiency of processes and optimizing resource allocation ( Suman, 2017).

## 4. Low-cost PTC options

The adoption of procedures and the use of equipment to minimize the unit cost of plantlets and overall plant output is known as inexpensive tissue culture technology. Low-cost alternatives should lower production costs without affecting micropropagule or plant quality. Cost savings are obtained with low-cost technologies by enhancing process efficiency and making better use of resources. In many developing nations, inexpensive tissue culture technology is a top goal in agriculture, horticulture, forestry, and floriculture to provide high-quality planting material at affordable prices ( George & Manuel, 2013).

Several low-cost alternatives can be used to simplify various operations and reduce costs in a tissue culture facility. The physical components of a typical PTC facility include equipment and buildings with a prep room, a transfer room, a culture or growing room, a hardening and settling area, a soil growing area (greenhouses, plastic tunnels), a packing and shipping area, and associated facilities such as a warehouse an office and chemical store, containers and supplies. The size of the physical components of a tissue culture facility depends on its functional requirements or production volume ( George & Manuel, 2013).

The costs of constructing and managing tissue culture facilities are high, and in developing nations, they are typically non-existent. Facility setup costs and unit production costs of micro propagated plants are high in developing nations, and the return on investment is typically disproportionate to the technology's potential economic benefits. Electricity, for example, is significantly cheaper in industrialized countries, and the supply is far more reliable than in underdeveloped countries. The same is true for micropropagation culture vessels, media, chemicals, equipment, and instruments. To minimize the cost of plant micropropagation, alternatives to expensive inputs and infrastructure must be identified and created ( Savangikar 2004; Purohit *et al*., 2011).

### 4.1 Low-cost options for surface sterilization

Several factors can influence the success of micropropagation, including the efficacy of surface sanitation, the type of culture medium used, and how browning symptoms are handled. As a result, explants must be handled aseptically with sterilized tools; nonetheless, disinfecting chemicals were previously utilized to destroy any germs that may have been present, particularly on the outside of the explants. The reasons of microbial contamination in tissue cultures have been explained in a variety of ways ( Abass, 2013). The approach utilized to sterilize the explants, instruments, and equipment could be one cause. In tissue culture, the most prevalent issues are improper procedures and insufficient disinfectant levels. External contamination of explants from contaminated tools, equipment, and personnel in media preparation and culture is also a hazard ( Abass, 2013; Rahayu *et al*., 2019; Emoghene *et al*., 2020).

One of the most important steps in PTC is surface sterilization of explants. Explants simply need to be surface sterilized by treatment with a disinfectant solution at acceptable concentrations for a defined amount of time; living materials should not lose their biological activity during sterilization, and only impurities should be removed; ( Oyebanji *et al*., 2009). Sodium hypochlorite (NaOCl), calcium hypochlorite Ca (ClO) _2_, mercuric chloride (HgCl _2_), silver nitrate (AgNO _3_), and hydrogen peroxide (H _2_O _2_) are some of the most commonly utilized sterilants ( Rahayu *et al*., 2019).

As a result, the sterilizing process is made simple, quick, and economical by using locally available bleach or berekina ( Oyebanji *et al*., 2009). As a result, standardizing both the concentration and exposure time for local bleach (berekina) for surface sterilization of sugarcane explants helps to sterilize explants at a lower cost and in a safer manner ( Mekonnen *et al*., 2013).

Topically prepared bleaching solution (JIC with 3.5% sodium hypochlorite) at time intervals between 20 and 45 minutes of surface sterilization of seeds and excised embryos of cowpea, rice, and sorghum explants resulted in a zero percent reduction in bacterial and fungal contamination ( Oyebanji *et al*., 2009). Additionally, sterilization of sweet potato nodule cuttings with JIK (commercial bleach) at 40% v/v containing 1.5% sodium hypochlorite for 20 min prevented microbial contamination ( Ogero *et al*., 2011). Similarly, Mekonnen *et al*. (2013) devised a safer and less expensive approach for surface sterilization of sugarcane explants using berekina instead of mercuric chloride as a surface sterilant. For surface disinfection of cane shoot tips, berekina with 5% chlorine active for 25 minutes of exposure was shown to be the best combination. Ayele & Tefera (2018) developed an inexpensive sterilization technique for the *in vitro* initiation of vanilla using the locally available sterilant berekina with 5% active chlorine treatment for 25 minutes and achieved an 82% contamination-free culture with a survival rate of 80% of plant regeneration.

The autoclaves (laboratory sterilizers) developed for laboratory sterilization applications make processes safer, simpler, more accurate, more reproducible, and more validatable. However, an electric autoclave is expensive; it requires a long heat-up time, electricity, special maintenance, and the risk of electrocution. Therefore, this can be replaced by pressure cookers, which are cheap, efficient, can use any heat source, are easy to maintain, and are safe by attaching a wire mesh to their base ( Ahloowalia & Prakash, 2004). Contamination is not detected when media and equipment have been sterilized using a pressure cooker ( Naik *et al*., 2020).

### 4.2 Low cost options for culture vessels

Several types of culture vessels are used in PTC culture laboratories, such as laboratory-specific culture jars, Erlenmeyer flasks, magenta bottles, or screw-capped test tubes and Petri dishes during the induction stage, but larger vessels were used when transferring explants for multiplication and elongation ( Ahloowalia & Prakash, 2004). Traditional tissue culture vessels may cost at least USD2.90 per piece. To reduce the costs in PTC, locally available glass jars that are used for food canning can be used in place of traditional tissue culture vessels. Hence, at least 85% of the cost could be reduced by using locally available jars. These potential alternatives can therefore find wide acceptability in developing countries that practice micropropagation ( Silvosa-Millado *et al*., 2020).

### 4.3 Bioreactor-based low-cost option for PTC

Bioreactors are vessels used to culture vast amounts of cells, tissues, or organs in liquid media. A growing variety of plants have shown numerous significant advantages over traditional semisolid micropropagation, including increased propagation rates and reduced space, energy, and labor costs. Increased interest in the usage of fluid systems, in general, in general, has been fueled by these cost-cutting benefits ( Levin & Tanny, 2004). As a result, when used in conjunction with a traditional laboratory, they can be appealing to developing countries looking to build new or expand existing plant culture facilities. Traditional culture tanks offer less precise control of plant growth, gas exchange, illumination, medium movement, temperature, and pH than bioreactors ( Adelberg, 2016).

The ability to scale up in a faster time frame, lower production costs, and automated control of physical and chemical conditions during the growth phase of PTCs is the main welfares of consuming a bioreactor culture method for propagation of economically significant plants ( Aladele *et al*., 2012). However, there are numerous drawbacks to using bioreactors in micropropagation due to a variety of issues. Contamination, a lack of procedures and production methods, increased hyperhydricity, foaming issues, shear stress, and the release of growth inhibiting chemicals from the cultures are just a few of the issues. Because liquid systems have more challenging contamination problems and a lack of problem-solving knowledge than semisolid growth media, they are more dangerous and demand more competence. As a result, in many impoverished nations, bioreactors are not a cost-effective solution. Labor expenses, production capacity, crops to be propagated, contamination rate, and energy and cost savings potential are all factors to consider ( Levin & Tanny, 2004; George & Manuel, 2013).

### 4.4 Low cost options for light

Light plays a crucial role in the growth and development of *in vitro* plant culture. It is a powerful source for photosynthesis and physiological processes to produce secondary metabolites. Among the many factors that contribute to plant growth, light is one of the most important obstacles to effective *in vitro* plant growth ( Vu *et al*., 2020). Typically, light intensity is set between 50 and 100 mol m ^-2^ s ^-1^ of photosynthetically active radiation, but photosynthetic photon fluxes as low as 5 or as high as 150 mol m ^-2^ s ^-1^ have been reported, depending on the species and application ( Phillips & Garda, 2019).

Because of the importance of light for plant growth, laboratories that affect plant tissues often consume a large amount of artificial light electricity. Artificial light generates heat that must be dissipated by cooling, and the fan further contributes to the electrical load. Therefore, switching the lighting method from artificial to natural light is a crucial cost-effective option in tissue culture. Light is a very expensive and ineffective method in tissue culture technology. In developing countries, electricity costs can account for up to 60% of production costs. In addition, its constant supply and power supply cause major problems ( George & Manuel, 2013; Vu *et al*., 2020).

While the need for electrical power is essential, adopting low-cost options can reduce production costs. The alternative of using natural light as a light source for micropropagation systems has been reported in various studies to reduce power and capital costs as well as to improve plant quality. For example, the use of light-emitting diodes in micropropagation can reduce electricity prices by 50 to 75% compared to traditional lighting systems. This is done by replacing artificial light with natural light ( George & Manuel, 2013; Vu *et al*., 2020).

### 4.5 Low-cost options for distilled water

Glass-distilled water is commonly used in tissue culture, with many laboratories using double distilled water ( Phillips & Garda, 2019). The use of ion-exchange columns has been criticized for the release of a variety of organic contaminants during the process ( True, 1914). However, ion exchange columns easily supply water to many laboratories and are widely used in Europe. For example, Ahloowalia and Prakash (2004) reported that double distilled water is the main component of all tissue culture media and is considered free of ions and impurities.

However, distilled water produced through electrical distillation is expensive and adds to the cost of tissue culture; hence, alternative, cheaper water sources that could reduce the cost of the media without altering the composition are needed. Tap water (free from heavy metals and contaminants) can be substituted for distilled water to lower the cost of the medium. Tap water after autoclaving can be used in small facilities rather than distilled water. Reverse osmosis water can be used for stock solutions and hormone preparations, and distilled water can be used for media preparation to reduce the cost most effectively. It is also used for washing plants before sterilization and for added sterilants for cleaning. Table bottled water from the supermarket can also be used as a low-cost alternative ( Naik *et al*., 2020). Rainwater, river water, and water from other natural sources were considered to be more beneficial to their experimental sites than distilled water, and natural waters were often used to ensure that plant behavior was considered normal ( True, 1914).

As a result, much research has been done to reduce the cost of double-distilled water. Raghu *et al.* (2007) reported that using tap water for tissue culture did not affect *in vitro* plant growth. Saraswathi *et al*. (2016) reported that for banana micropropagation, three types of water (reverse osmosis water, drilled well water, and simple distilled water) were evaluated to reduce media preparation costs. Water costs were reduced by 86% by using inexpensive replacement water (reverse osmosis water, artesian well water) instead of double-distilled water. Similarly, Sunandakumari *et al*. (2004) reported that media prepared with tap water differed from media prepared with double distilled water in terms of *in vitro* sprout induction in *Mentha piperita.*

### 4.6 Low-cost options for media components

For semisolid and solid media, a typical PTC medium includes a base solution containing major and minor minerals, a carbon source (sucrose), vitamins, growth regulators, and a gelling agent ( Bhojwani & Dantu, 2013). However, there is an ongoing search for alternatives for the culture medium components to lower plant micropropagation expenses. The major goal is to substitute nutritive solutions derived from less expensive sources for macro- and micronutrients, sugars, and gelling agents ( Montenegro *et al*., 2014). These are the key variables that contribute to the high production costs.

We can considerably lower the cost of tissue culture by adopting low-cost alternatives, allowing it to be practiced by the common farmer. Using low-cost alternatives, tissue culture plant manufacturing costs can be decreased by 50-90 percent. Foliar nutrients have been used in the in vitro culture of *Cattleya* sp., seed culture of Arabidopsis thaliana, fertilizers for *Laelia anceps* micropropagation, and numerous commercial foliar fertilizers in the micropropagation of *Solanum tuberosum* have been reported ( Montenegro *et al*., 2014).

The gelling agent is another ingredient of PTC media. Agar is one of the most commonly used gelling agents in the preparation of culture media, but it is also one of the most expensive. Alternatives such as cassava starch, wheat flour, semolina, potato starch, rice powder, sage, bulla, and others have allegedly been used to generate inexpensive and accessible media for commercial *in vitro* plant production ( Mohamed *et al*., 2010). In the case of the root-initiating MS medium, some other ingredients, such as sawdust, peanut hulls, rice husks, coconut pulp, and coir, were used instead of agar ( Das & Gupta, 2009).

**4.6.1 Low-cost options for gelling agent**

Culture growth and shoot or root formations are greatly affected by the physical instability of the medium. Gelling agents are often added to the medium to increase its viscosity, thereby keeping the tissues of plants and organs above the surface of the medium ( Ahloowalia & Prakash, 2004). The rigidity of the medium greatly affects the growth of the cultured tissue. A wide range of gelling agents used in plant culture media are available on the market, including agar, agarose, and gellan gum sold under trade names such as Phytagel, Gelrite, and GelGro ( Ahloowalia & Prakash, 2004; Franklin & Franco, 2021).

Agar has been widely used as a medium for microorganisms and PTC since it was introduced as a gelling agent over 100 years ago. It is a polysaccharide derived from red algae and the family Rhodophyceae. It is used as a universal gelling agent for preparing semisolid and solid media for PTC. The concentration of agar used in the medium depends on its purity and productivity. It is usually applied at 0.6-0.8% (w/v) ( Franklin & Franco, 2021). It is the most expensive component of the PTC medium and usually contributes to increased medium viscosity at 70% of the medium cost ( Naik *et al*., 2020). The almost exclusive use of agar leads to overfishing of its source. Due to the exorbitant price of tissue culture grade agar, attempts have been made to find suitable alternative gelling agents that are economically feasible and readily available locally ( Khan *et al*., 2012).

Recently, various inexpensive sources, such as potato flour, rice, barley, wheat, enset flour/bulla, and corn flour, have also been used individually or in combination as gelling agents with varying degrees of success ( Mohamed *et al*., 2010; Ayenew *et al*., 2012; Khan *et al*., 2012). It has also been found that the combination of laundry starch, potato starch, and semolina in a 2:1:1 ratio reduces the cost of the gelling agent by more than 70% ( Prakash, 1993).

**Bulla**: Bulla is a processed starch or dough from Enset. It is a byproduct of making kocho when the waste and pulp are pressed, separating the liquid. The starch that separates from the liquid concentrates into a white powder.

Bulla was first used for the micropropagation of pineapple (Ananas comosus) in place of agar at 80 g/L. The results showed that production costs were reduced up to 76% ( Ayenew *et al*., 2012). Another report from the same year found that replacing agar with 60-100 g/L of bulla could reduce the cost of micropropagation of vanilla planifolia by 50-72.5% ( Mengesha *et al*., 2012). Similarly, for cassava meristems, culture bulla at 80 g/L reduced costs by 86% when combined with 60 g/L bulla + 2 g/L agar, and 70 g/L bulla + 1 g/agar saved a cost of 65-75% ( Ayalew *et al*., 2017).

In addition to replacing traditional agar, bullas have been found to increase root length compared to traditional agar. This may be due to the carbon content. However, bulla is less transparent, and contamination is difficult to detect ( Ayenew *et al*., 2012; Mengesha *et al*., 2012; Ayalew *et al*., 2017). Similarly, other types of starches may exhibit such greening or darkening properties, e.g., Saraswathi *et al*. (2016) reported that three gelling agents were used, and greening (10%-20%) was observed in a medium with corn flour.

**Isubgol**: Isubgol is extracted from the seeds of the *Plantago ovata* of the Plantain family. The effect of Isubgol's skin is solely due to the presence of large amounts of mucus in the skin. Similar to agar, ibugor mucus is colloidal and polysaccharide-like, consisting primarily of xylose, arabinose, galacturonic acid, rhamnose, and galactose. The slimy husks from Plantago ovata have been successfully used as a gelling material for tissue culture media. The price of “Isubgolhusk” is cheaper than that commonly used as agar because the price of the gelling agent in the PTC medium has been reduced by approximately 47.5% ( Khan *et al*., 2012).

This has been evidenced by different previous reports. For instance, replacing the commonly used solidifying agent agar (0.8%) with Isbugor (1.5%) produced a much similar response to agar for callus formation in sugarcane *in vitro* propagation ( Dhawale *et al*., 2021). In another report by Ullah *et al*. (2015), isabgol at 30 g/L was the optimal cost-effective gelling agent for the best growth of shoots, leaves, and roots of orchids (Dendrobium Sonia). Similarly, Isabgol-based medium enhanced the initial establishment of shoot tips in ‘Udhayam’ and ‘Rasthali’ banana cultivars, and the combination of Sago and Isabgol was found to be a better gelling agent for many crops, including banana. The effectiveness of these alternative gelling agents might have been due to their high mucilage content (>30%) or their polysaccharide and colloidal nature ( Saraswathi *et al*., 2016).

**Potato**, **barley**, and **corn starch**: Starch is the cheapest alternative among the alternative gelling agents in PTC, and its use may reduce costs. Nevertheless, starch is hydrolyzed by plant amylolytic enzymes during *in vitro* tissue culture ( Lima *et al*., 2011). To evaluate this situation, Amlesom *et al.* (2021) examined three alternative low-cost corn, potato, and barley starches at 50 g/L as a solidifying substitution for commercial agar for the micropropagation of potato. It has been found that substituting agar with alternative starches showed a significant reduction in the cost of a solidifying agent. The highest cost reduction was observed from 61-66%, implying that using both corn and potato starches can be reliable and cost-effective gelling agents for the micropropagation of potatoes.

**Sago** starch: Sago starch is a complex polysaccharide that serves as a storage product in a variety of plants. It contains a small number of sugars, fiber, protein, calcium, and other minerals. Sago, unlike agar, can be used as a gelling agent and as a carbohydrate component in nutrient media ( Datta *et al*., 2017).

Sago, as a gelling agent, had a significant influence on shoot proliferation and *in vitro* rooting of ginger, turmeric, and banana. Sago at 70 g/L gave proper solidification and normal culture growth in ginger and turmeric micropropagation ( Prakash, 1993). Similarly, medium solidified with sago at 80 g/L was found to be superior to commercial grade agar for the micropropagation of banana in terms of shoot length, several leaves, shoot diameter, several primary roots, plantlet diameter, and fresh weight, and it reduced the media cost by 69.69% ( Prabhuling, *et al*., 2014). However, beyond the better performance of sago in the medium, it may reduce visibility to check for fungal growth under *in vitro* conditions ( Saraswathi *et al*., 2016).

Additionally, the utilization of glass beads has potential. Glass beads provide a good substratum for plant tissues that grow well in a liquid media, with the added benefit of reuse, lowering the input cost by removing the use of a solidifying agent in every cycle ( Pant, 2016).

**4.6.2 Low-cost options for macro- and micro-nutrients**

Plant tissue and organs produced in vitro on synthetic media provide the nutrients required for growth and development. Plant propagation via tissue culture is impacted by the type of media utilized. Because plants require considerable amounts of inorganic elements, they are referred to as major plant nutrients. N, P, Ca, P, Mg, and S are the main elements. Micronutrients, on the other hand, are elements that are required in little amounts (Fe, Cu, Zn, B, Mo, Mn, and Cl) ( Purohit *et al*., 2011; George & Manuel, 2013; Ayalew *et al*., 2017; Phillips & Garda, 2019).

Locally available fertilizers at appropriate concentrations can be used as a low-cost source of nutrients for PTC. The conventional sources of MS media were replaced by mixed nutrients containing both macro- and micronutrients for sweet potato micropropagation. MS nutrients were substituted with locally available fertilizers as a macronutrient (monopotassium phosphate, potassium fertilizer, Epsom salt, and ammonium quarry salt) and micronutrients (Stanes Iodised Microfood ^®^) using 30 g/L table sugar as a source of carbon. The low-cost medium was significantly cheaper than the MS medium, with reductions of 87.8%, 68.6%, and 97.1% for macronutrients, micronutrients, and carbon sources, respectively ( Ogero *et al*., 2011). Ogero *et al*. (2012) found that replacing MS salt medium with locally available foliar feed “Easygro vegetative fertilizer” comprising both macro- and micronutrients resulted in a low-cost cassava micropropagation strategy. The usage of Easy Grows led in a 96.7% cost decrease. Similarly, alternative sources to MS macronutrients included ammonium fertilizer, potassium fertilizer, Epsom salt, monopotassium phosphate, and calcination, while Stanes Iodized Microfood ^®^ was used as an alternative to MS micronutrients for cassava micropropagation, resulting in a 93% cost savings ( Kidulile *et al*., 2018).

Hydro Agri’s fertilizer is also another alternative option to replace MS media salts for micropropagation of cassava, and the full substitution of the media with commercially available nutrients resulted in a cost reduction of 93.1% compared to the traditional media ( Naik *et al*., 2020). In addition, palm oil mill effluent is a free and nontoxic material that contains valuable macronutrients required for plant growth. It consists of high total nitrogen sources that increase the composting period efficiency by degrading the cellulose and hemicellulose components of empty fruit bunches. In addition, the organic nutrient constitution in palm oil mill effluent and empty fruit bunch is desirable by the plant, which can reduce the usage of inorganic fertilizer as part of environmental protection. A 100 mL palm oil mill effluent solution with 100 mL molasses was found to be more cost-effective than the synthetic MS basal salt medium. Thus, this will also minimize the production cost of the clonal plant without compromising the quality of regeneration produced ( Nadirah *et al*., 2019).

**4.6.3 Low-cost options for carbon source**

In vitro, plant cells, tissues, and organ cultures are not fully autotrophic, necessitating the use of carbohydrates in culture media to maintain osmotic potential and provide energy and carbon for developmental processes such as shoot proliferation, root induction and emission, embryogenesis, and organogenesis, all of which are energy-intensive developmental processes in plant biology ( Yaseen *et al*., 2013).

Therefore, the addition of suitable carbonaceous materials as an energy source is imperative. In plant cell culture media, sucrose is mainly used as an energy source. Sucrose is partially broken down into fructose and glucose during autoclaving. Sucrose also acts as an osmoticum in the medium. Generally, 20-40 grams of sucrose is added to each liter of liquid medium. Starches are also used in some medium formulas both as the support substance and energy source. Sucrose has been reported to act as a morphogenetic trigger in the formation of accessory buds and branching of adventitious roots ( George *et al*., 2008). Consequently, sucrose is among the media components that are routinely used in PTC. However, the unavailability and the prohibitive cost of laboratory-grade sucrose are among the major constraints of PTC laboratories found in developing countries ( Rukundo *et al*., 2012).

As a result, instead of tissue culture-grade sucrose, commercially available table sugar was employed. This resulted in lower medium costs without sacrificing micropropagation rate or plant quality ( Venkatasalam *et al*., 2013). Furthermore, adding cane molasses, banana extract, and coconut water to basal media has been discovered to be a suitable choice for lowering medication prices. These substrates provide not only energy but also vitamins and inorganic ions, which are essential for growth.

Alternative energy sources for micropropagation of various plant species have been documented in numerous researches. Using locally available alternatives the energy cost for potatoes TC was reduced by 34 to 51% ( Demo *et al*., 2008). Table sugar at 30 g/L could stimulate banana plantlet growth to the same extent as laboratory sucrose at 30 g/L. As a result, table sugar is advised for banana micropropagation to lower the cost of in vitro plantlets. Plantlets grown on table sugar culture medium, on the other hand, were shown to be weaker than counterparts grown on laboratory grade sucrose culture medium ( Rukundo *et al*., 2012).

Mekonnen *et al*. (2014) developed an inexpensive protocol that can use table sugar 6% half MS for rooting and 4% table sugar for sprout propagation instead of expensive sucrose 6% with half MS and 3% sucrose for sprout propagation. Therefore, to let developing countries such as Ethiopia benefit from PTC technology, using such locally available and economically justifiable resources instead of expensive resources is the best alternative. Identifying cheap or low-cost alternative gelling and carbon sources will significantly reduce production costs by 90-97% ( Mengesha *et al*., 2021; Datta *et al*., 2017). In addition, using vermicompost 50 g/L+ table sugar 30 g/L, wheat flour, and coconut water reduces production costs by 58 to 97% ( Kadam *et al.*, 2018; Rasal-Monir, 2018).

## 5. Conclusion

PTC is considered to be a mass propagator of agriculturally important crops within a finite period regardless of the season. However, its application is hampered by the costs required to carry out the activities. Therefore, various developing countries, including Ethiopia, suffer from the high costs of media, facilities, and skilled labor, as well as adequate funding, staff turnover, and government policies, which pose the greatest challenges for PTC in developing countries. To meet these challenges, various scientists have searched for solutions in the past. One solution that scientists are recommending for developing countries to use PTC applications is to replace low-cost media alternatives, gelling agents, locally available culture vessels, natural light, tap water, table sugar as a carbon source, and others. Furthermore, the government should pay attention to adequate funding for the PTC sectors in the country, train employees, provide incentives to reduce staff turnover, and focus on expanding laboratory facilities to promote the technology and use it effectively.

## Data availability

There are no underlying data associated with this article.
