# Supplementary material for: Plant tissue culture challenges in Ethiopia and alternative options for low-cost media
Source: F1000Res. 2022 Jul 26;11:828. [Version 1] doi: 10.12688/f1000research.122627.1 (PMC11406133; doi:10.12688/f1000research.122627.1)
Supplement: Peer review history

# Reviewer report: Reviewer response for version 1

**DOI:** 10.5256/f1000research.134646.r215945

**Published:** 2024-09-16

**Authors:** Jaindra Nath Tripathi

**Affiliations:** 1 International Institute of Tropical Agriculture (IITA), Nairobi, Kenya

The manuscript is well written, focusing on plant tissue culture challenges in Ethiopia and suggesting how to reduce the cost using alternative options. I agree with the authors to use low-cost options to reduce the cost. it is very difficult to buy all the tissue culture consumables from international companies in developing countries like Ethiopia and other African countries.

Is the review written in accessible language?

Yes

Are all factual statements correct and adequately supported by citations?

Yes

Are the conclusions drawn appropriate in the context of the current research literature?

Yes

Is the topic of the review discussed comprehensively in the context of the current literature?

Yes

Reviewer Expertise:

Plant tissue culture and Plant Transformation, Genome editing,

I confirm that I have read this submission and believe that I have an appropriate level of expertise to confirm that it is of an acceptable scientific standard.

# Reviewer report: Reviewer response for version 1

**DOI:** 10.5256/f1000research.134646.r228304

**Published:** 2024-02-16

**Authors:** Dr. Nurul Izzati Osman

**Affiliations:** 1 Faculty of Pharmacy, Universiti Teknologi MARA Cawangan Selangor, Selangor, Malaysia

(1) Suggestion: Concrete reference/supporting studies are required for certain claims/statements in the manuscript, for example:

 -"University graduates (Bachelor) do not want to work as normal workers in laboratories".

 -"Furthermore, adding cane molasses, banana extract, and coconut water to basal media has been discovered to be a suitable choice for lowering medication prices "

 (2) Some of the references cited don't reflect the statement mentioned in the manuscript, for example:

 -"In other cases, alternative options for culture media, such as cane molasses, cane juice, banana extract, and coconut water, are good alternatives to high-quality sucrose to reduce mean costs ( Getnet *et al*., 2016)".

 Getnet et al., 2016 research is on table sugar, not cane juice, banana extract etc.

 (3) Delete repeated words: "Increased interest in the usage of fluid systems, in general, in general, has been fueled by these cost-cutting benefits..."

 (4) Suggestion: Incorporate latest/recent findings and references for classic reference, for example:

 "Rainwater, river water, and water from other natural sources were considered to be more beneficial to their experimental sites than distilled water, and natural waters were often used to ensure that plant behavior was considered normal ( True, 1914)."

 (5) For this study, what was the impact on micropropgated plants? The implication for the quality of yields and products must be highlighted.

 "Water costs were reduced by 86% by using inexpensive replacement water (reverse osmosis water, artesian well water) instead of double-distilled water."

 (6) Why adequate infrastructure is a serious bottleneck?: "Ethiopian government-owned PTC laboratories are **characterized by adequate infrastructure, and these are a serious bottleneck **..."

 (7) No space between Ca and (ClO)2. Please check.

 (8) Revise the sentence:

 "Explants simply need to be surface sterilized by treatment with a disinfectant solution at acceptable concentrations for a defined amount of time; living materials should not lose their biological activity during sterilization, and only impurities should be removed; ( Oyebanji *et al*., 2009)."

 (9) Revise the sentences. Twice 'As a result' in a consecutive order in one paragraph. 

 " **As a result, **the sterilizing process is made simple, quick, and economical by using locally available bleach or berekina ( Oyebanji *et al*., 2009). **As a result**, standardizing both the concentration and exposure time..."

 (10) There is a reference that cannot be accessed: Abbebaw et al. 2021. Is it similar to Abebaw et al. 2021?

 (11) Suggestion: It is good if the facts and figures regarding the demand for PTC-produced plants in Ethiopia are included in the review, and how many are successfully met with the current status of PTC in Ethiopia to depict the challenges of PTC.

 General comment: Please check the sentence structures of the whole manuscript, the currentness, and suitability of the references cited.

Is the review written in accessible language?

Partly

Are all factual statements correct and adequately supported by citations?

Partly

Are the conclusions drawn appropriate in the context of the current research literature?

Yes

Is the topic of the review discussed comprehensively in the context of the current literature?

Yes

Reviewer Expertise:

Plant Tissue Culture, Phytochemistry, Pharmacognosy

I confirm that I have read this submission and believe that I have an appropriate level of expertise to confirm that it is of an acceptable scientific standard, however I have significant reservations, as outlined above.

# Reviewer report: Reviewer response for version 1

**DOI:** 10.5256/f1000research.134646.r204125

**Published:** 2023-09-25

**Authors:** Marimuthu Somasundaram Saraswathi

**Affiliations:** 1 ICAR-National Research Centre for Banana, Trichy, Tamil Nadu, India

Though the low cost options have been covered comprehensively in this review, it needs revisions in the following areas.

- In all places, wherever you have mentioned about cost reduction, it should be in terms of % reduction as against control or the standard practice.
- The scientific names should always be in italics.
- The term *in vitro* should be italicised.
- It is very poorly written, they are advised to proofread the article.
- References
  Franklin and Franco, 2021 in page No.10 4.6.1. last but one line. In the references, the retrieved date is not required as it cause confusion of 2022 or 2021
  Abebaw et al. (2021) is the right spelling and in Page 3, 4 ^th^ para last line it has been wrongly spelled as Abbebaw. Hence this may be corrected please.
  References formatting is not uniform, this may be re-checked please.

Please find an annotated pdf with further corrections here.

Is the review written in accessible language?

Partly

Are all factual statements correct and adequately supported by citations?

Yes

Are the conclusions drawn appropriate in the context of the current research literature?

Yes

Is the topic of the review discussed comprehensively in the context of the current literature?

Partly

Reviewer Expertise:

Tissue culture, mutation breeding, molecular biology and genetic resources management

I confirm that I have read this submission and believe that I have an appropriate level of expertise to confirm that it is of an acceptable scientific standard, however I have significant reservations, as outlined above.
